# Supplementary figures and images for: Reproductive strategy of Delta Smelt Hypomesus transpacificus and impacts of drought on reproductive performance
Source: PLoS One. 2022 Mar 10;17(3):e0264731. doi: 10.1371/journal.pone.0264731 (PMC8912181; doi:10.1371/journal.pone.0264731)

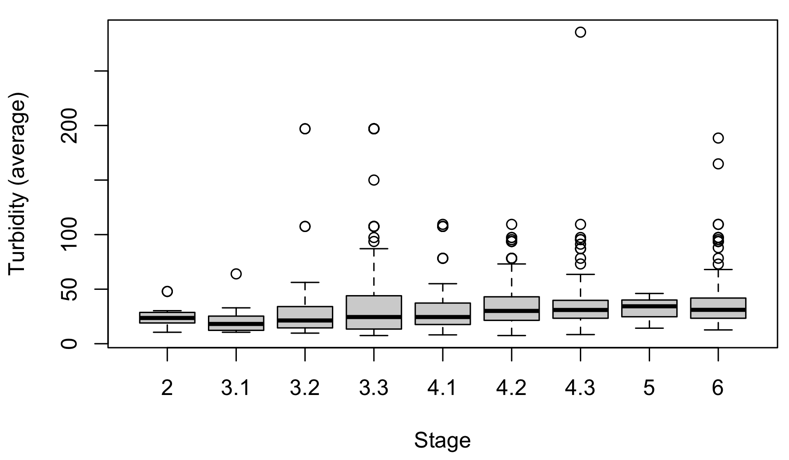

Supplement: S1 Fig — Maturity of female fish was scored based on the gonadal histological features [38]. (TIFF) [file pone.0264731.s001.tiff]

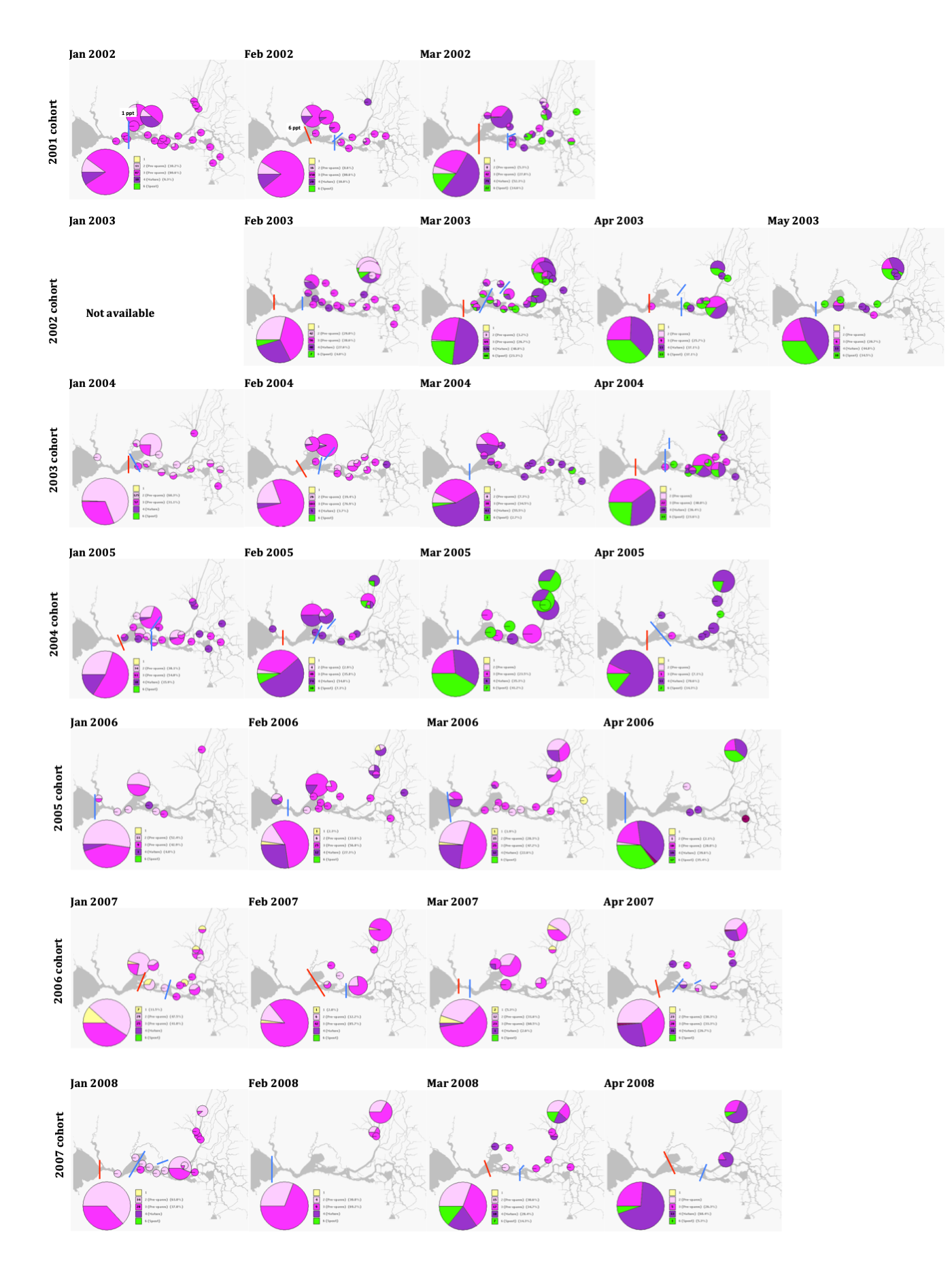

Supplement: S2 Fig — The data were obtained from the California Department of Fish and Wildlife website (https://www.wildlife.ca.gov/Conservation/Delta/Spring-Kodiak-Trawl). The blue and red lines indicate salinity boundary for 1.0 and 6.0, respectively. (TIFF) [file pone.0264731.s002.tiff]

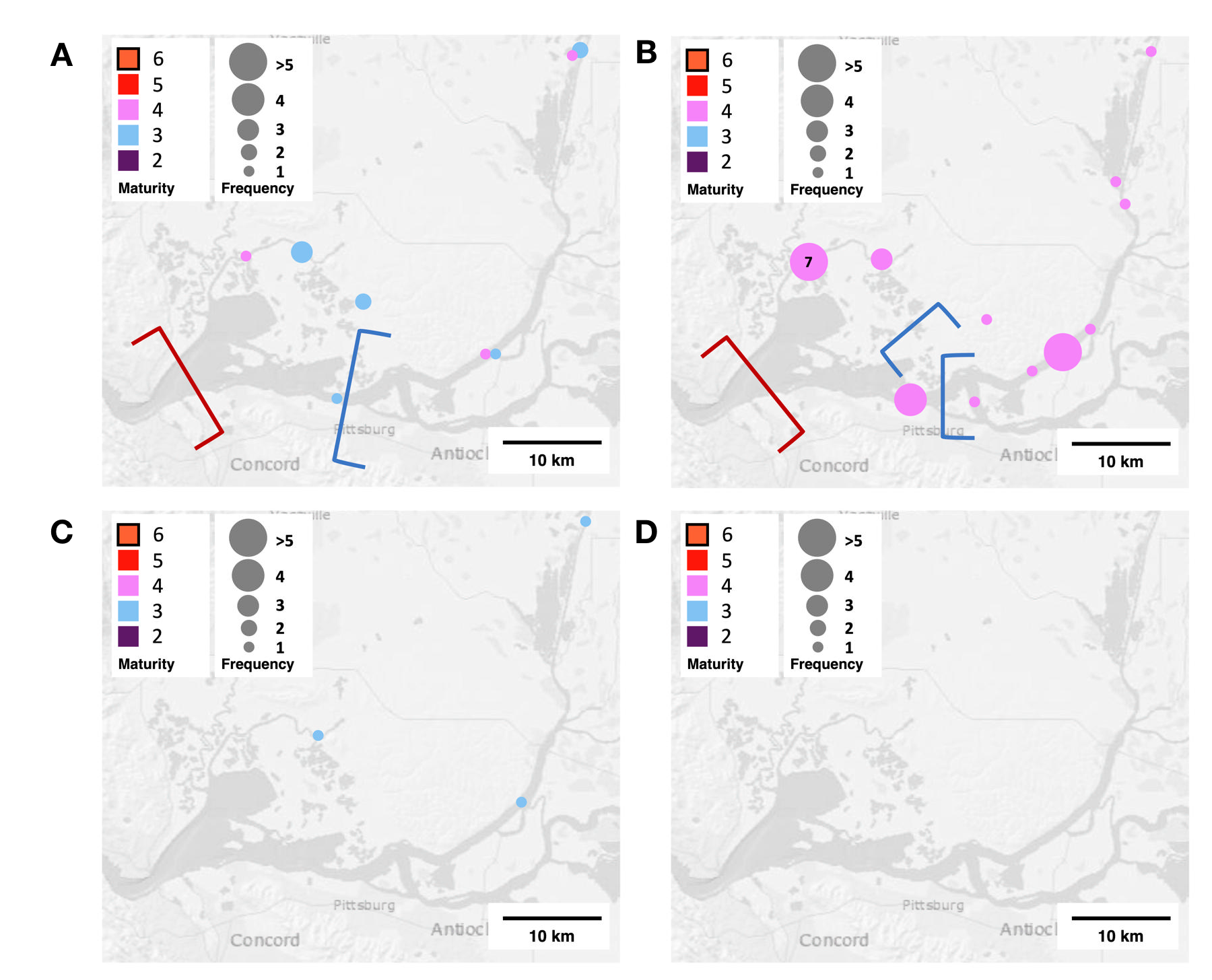

Supplement: S3 Fig — (TIFF) [file pone.0264731.s003.tiff]

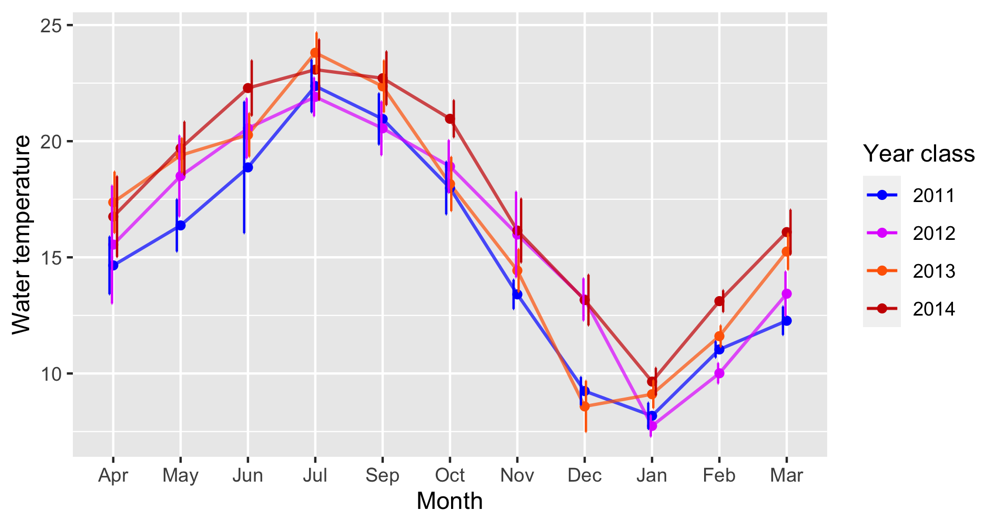

Supplement: S4 Fig — The water temperature data were obtained from the California Department of Fish and Wildlife FTP server (ftp://ftp.wildlife.ca.gov/; 20mm Survey, Fall Midwater Trawl Survey, and Spring Kodiak Trawl Survey). The August data are not available. Mean water temperatures of the four regions (Suisun Marsh and Montezuma Slough, Confluence of the Sacramento and San Joaquin rivers, Sacramento River, and Cache Slough Complex) are depicted for each month. Stations with salinity higher than 6 are not included. The error bars indicate standard deviation. (TIFF) [file pone.0264731.s004.tiff]
